# Rates of Molecular Evolution in a Marine *Synechococcus* Phage Lineage

**DOI:** 10.3390/v11080720

**Published:** 2019-08-06

**Authors:** Anne Kupczok, Tal Dagan

**Affiliations:** Genomic Microbiology Group, Institute of General Microbiology, Christian-Albrechts University, 24118 Kiel, Germany

**Keywords:** substitution rate, recombination rate, gene turnover, cyanophage

## Abstract

Cyanophages are characterized by vast genomic diversity and the formation of stable ecotypes over time. The evolution of phage diversity includes vertical processes, such as mutation, and horizontal processes, such as recombination and gene transfer. Here, we study the contribution of vertical and horizontal processes to short-term evolution of marine cyanophages. Analyzing time series data of *Synechococcus*-infecting *Myoviridae* ecotypes spanning up to 17 years, we found a high contribution of recombination relative to mutation (r/m) in all ecotypes. Additionally, we found a molecular clock of substitution and recombination in one ecotype, RIM8. The estimated RIM8 evolutionary rates are 2.2 genome-wide substitutions per year (1.275 × 10^−5^ substitutions/site/year) and 29 genome-wide nucleotide alterations due to recombination per year. We found 26 variable protein families, of which only two families have a predicted functional annotation, suggesting that they are auxiliary metabolic genes with bacterial homologs. A comparison of our rate estimates to other phage evolutionary rate estimates in the literature reveals a negative correlation of phage substitution rates with their genome size. A comparison to evolutionary rates in bacterial organisms further shows that phages have high rates of mutation and recombination compared to their bacterial hosts. We conclude that the increased recombination rate in phages likely contributes to their vast genomic diversity.

## 1. Introduction

Interactions with (bacterio-)phages constitute a major determinant of bacterial evolution and ecology. First, differential phage predation modulates bacterial population structure. This is especially well documented in the marine environment [1,2], where bacterial cell lysis during phage infection is crucial for nutrient recycling in marine biogeochemical cycles [3,4,5]. Second, phage-mediated gene transfer can facilitate the adaptation of marine bacteria to specific habitats or lifestyles [6]. In addition, auxiliary metabolic genes (AMGs) in phage genomes supplement the host metabolism during lytic infection. This is especially well documented for phages that infect cyanobacteria (e.g., [5,7,8]), where genes that encode for proteins in the photosynthesis pathway have been acquired by phages [9].

The rate of phage evolution is thought to be mainly driven by antagonistic coevolution of phages with their bacterial host [10,11]. Phage genome evolution comprises vertical and horizontal processes. The ultimate source of genetic variation during evolution is mutation of the DNA. Mutation rates have been estimated, by fluctuation tests, to be between 2 × 10^−8^ and 8 × 10^−7^ mutations per nucleotide per cell infection for dsDNA bacteriophages and about 10^−10^ mutations per nucleotide per replication for bacteria [12,13]. In contrast, the nucleotide substitution rate measures the number of mutations that persist in the population over time. It can be estimated from time series samples of a ‘measurably evolving population’, which is characterized by a sufficient sampling time span, the number of samples, and a fast substitution rate relative to the sampling time span [14]. To estimate if the sampling time span is appropriate for substitution rate estimation, one has to test for the presence of a temporal signal. This can be done by randomizing the sampling times and comparing the parameter estimates between the original and the randomized data sets [15]. Substitution rate estimation for measurably evolving microbial populations has previously focused on pathogenic organisms. These analyses showed that numerous species of bacterial pathogens exhibit a strong temporal signal [16] and yielded substitution rate estimates between 10^−8^ and 10^−5^ substitutions per site per year [17]. A similar approach has been used to study the evolution of dairy *Siphoviridae* phages, which revealed a high temporal signal and a substitution rate of ~2 × 10^−4^ substitutions per site per year [18]. The degree to which this estimate reflects the evolutionary dynamics in other phage lineages is currently unknown.

In addition to mutation, bacterial and phage genomes also evolve by horizontal processes. The acquisition of DNA from other individuals has the potential to alter multiple nucleotides in a single event (termed here recombination), or it can result in the acquisition of a new gene and, consequently, in gene content variation over time (termed here gene gain and loss or gene turnover). Horizontal evolution in phages depends on the frequency of co-infection, i.e., the infection of a bacterial cell by two viruses at the same time. Co-infection has been shown to be prevalent in nature [19]. For example, a study of marine SUP05 bacteria revealed that ~35% of the infected bacteria are co-infected by more than one phage [20]. The impact of co-infection frequency on the rate of horizontal evolution in phages remains understudied.

T4-like cyanomyoviruses (termed here cyanophages) that infect marine *Synechococcus* and *Prochlorococcus* are abundant in the ocean and their genome is characterized by a stable core genome and hyperplastic regions [21,22]. Genetic mechanisms that facilitate homologous recombination have been well investigated in T4, where DNA recombination, replication, and repair pathways are closely linked [23]. The importance of recombination in cyanophage evolution was also shown in the analysis of eight genes from closely related cyanophages, which revealed the absence of linkage disequilibrium and several events of intragenic recombination [24]. Intragenic recombination also affects the evolution of AMGs, such as *psbA* and *psbD* [24,25]. Recombination was additionally hypothesized to mediate the acquisition of foreign genes, including AMGs [25,26]. In archaea and bacteria, boundaries of gene flow in recombining populations result in the formation of cohesive genomic units or ecotypes [27]. Recently, such ecotypes have also been observed in cyanophages, where ecotypes at the level of core genome divergence are linked to differences in accessory gene content, presumably including niche-differentiating genes [28,29]. This suggests that barriers to gene flow also contribute to the evolution of ecotypes in phages [30,31].

Here, we study phage genome evolution using the cyanophage data set of Marston and Martiny [29], which comprises complete genomes of *Synechococcus*-infecting cyanophages that have been sampled over 15 years. Genomic diversification within ecotypes has been described to be affected by single-nucleotide polymorphisms, recombination, and gene gain and loss [29]. However, the relative contribution of each of these processes and their temporal dynamics remain unknown. Here, we analyze the vertical and horizontal evolutionary processes in these ecotypes, assess their temporal signal, and estimate evolutionary rates.

## 2. Materials and Methods

We retrieved the cyanophage data set of Marston and Martiny (described in [29], list of accessions available in Table S2 [29]) and four additional recently released genomes for RIM8 (GenBank accessions MK493322, MK493323, MK493324, MK493325). The genomes have been grouped using average nucleotide identity (ANI) into clusters and subclusters [29]. Here, each cluster or subcluster with at least six genomes available has been analyzed independently. ProgressiveMauve v2.4.0 [32] with the option --seed-family confirmed that the set of genomes for each ecotype is collinear. A whole-genome alignment was obtained using MAFFT v7.123b [33] with the --auto option. Maximum likelihood (ML) phylogenies were estimated using IQ-TREE v1.6.8 [34] with the best substitution model selected using ModelFinder [35]. Recombination events were detected with ClonalFrameML v1.25 [36] based on the ML phylogeny and the respective kappa estimated under the HKY model with IQ-TREE. ClonalFrameML detects recombination events on the branches of the given phylogeny by also considering its branch lengths. Recombined segments detected with ClonalFrameML are characterized by the start and end position in the alignment and the branch in the phylogeny where the segment is introduced. Recombinations were masked in an alignment by masking recombinant positions detected by ClonalFrameML. Thereby, recombinant segments on terminal lineages were replaced by gaps and recombinant segments on internal lineages resulted in masking of the whole alignment region. The ML phylogeny was re-estimated for the masked alignment. This might result in a different topology and different branch lengths estimates. As ClonalFrameML detects recombination events on the branches, it might be able to detect further events with the new phylogeny. The process of recombination detection, masking, and phylogeny estimation was repeated until no more recombined segments were detected. This procedure is expected to converge to the clonal phylogeny of the lineage. However, recombination events can only be detected if they introduce multiple differences. Thus, events which introduce only one or few differences may remain undetected; nonetheless, such differences can introduce conflicting phylogenetic signal. To test for the impact of such undetected recombination events, we applied the Phi test as implemented in SplitsTree4 [37], which tests the null hypothesis of no recombination in the alignment [38].

Evolutionary rates were estimated using least-squares-dating (LSD v0.2) [39] with the options –c –r a -t 1e-10 (date constraints on the nodes, search for the root on all branches, and minimum rate of 10^−10^ substitutions/site/year). Substitution rate estimation with this distance-based method was chosen because it has been shown to be robust to model assumptions such as the strict molecular clock [39]. Substitution rates were estimated from the ML tree of the masked alignment. Recombination rates were estimated from a phylogeny, where branch lengths represent the number of recombination events, the total recombination segment length transferred, or the number of nucleotide alterations (see Reference [18] for details). LSD reconstructs the evolutionary rate and a dated phylogeny. The similarity of the raw branch lengths (i.e., the branch lengths of the LSD input) and the dated branch lengths (i.e., the branch lengths of the dated phylogeny) was estimated by the cosine similarity, which is independent of the vectors’ magnitudes. The temporal signal was assessed by resampling the dates 100 times and re-estimating the evolutionary rates. An additional measure of the temporal signal was the correlation between the root-to-tip (RTT) distance with the isolation date, as calculated with treetime v0.2.4 [40].

Proteins were classified into the same homologous protein family if they had a blastp e-value <10^−10^ (BLASTP+ v2.4.0) and a global identity larger than 60%. Protein families were assigned to be core families if they were present in each genome and variable families otherwise. The 35 protein families described as T4 phage core genes [30] were mapped to the protein clusters as follows. The best blastp hit of KX349285 to T4 (RefSeq accession NC_000866) was found and the protein family which contained a hit to a T4 phage core gene was assigned to be a T4 phage core family. Of the 35 families, 33 had an e-value < 0.01 and are considered here as T4 phage core genes. Notably, these correspond to the 33 T4 phage core genes listed in Reference [29]. The position of each gene family was mapped to the whole-genome alignment by detecting the smallest start position and the largest end position of each gene family member in the alignment. Homologs in other cyanophages were found by blastp against nr with an e-value <10^−10^.

## 3. Results

### 3.1. Recombination Detection

For the study of phage genome evolution, we analyzed nine cyanophage clusters and subclusters [29] (Table 1). To discriminate the contribution of mutation and recombination, we first applied ClonalFrameML iteratively to mask recombined segments and subsequently tested for the presence of recombination with the Phi test. Applying ClonalFrameML, we found that the contribution of recombination relative to mutation to the nucleotide differences (r/m) ranges between 0.3 and 15, with eight of the nine lineages showing an r/m value larger than one. This indicates that the contribution of recombination exceeds the contribution of mutation in most of the analyzed lineages. We observe that six of the nine alignments still contain a signal for recombination after masking (*p*-value Phi test < 0.01), which denotes that detecting recombined segments with ClonalFrameML is not sensitive enough to mask the conflicting information from the alignments. As the absence of recombination is a prerequisite for the evolutionary rate analysis, the following analysis is restricted to RIM8, RIM12_C, and RIM44, where the masking eliminated the signal of recombination.

### 3.2. Temporal Signal and Substitution Rate Estimation

**Figure 1:**
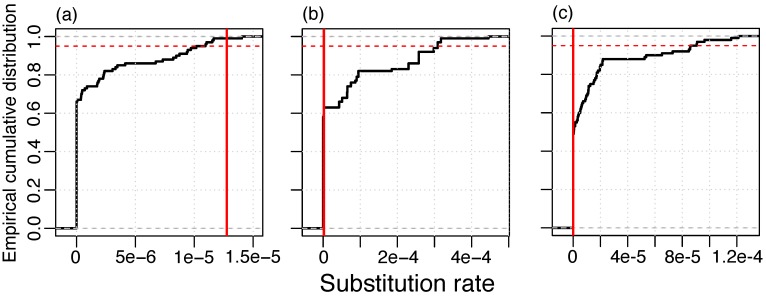
Resampling test for temporal signal of substitution in the masked alignment; (**a**) RIM8, 1.275 × 10*^−^*^5^ substitutions/site/year; (**b**) RIM12_C, 2.070 × 10*^−^*^6^ substitutions/site/year; and (**c**) RIM44, 10*^−^*^10^ substitutions/site/year. Vertical red line: Estimate of the real data set. 95% of the resampled data sets lie below the red dashed line.

To determine whether substitutions in the phage lineages contain a temporal signal, we applied a resampling method where dates were randomly re-assigned to genomes in the masked alignment and rates are estimated with LSD. In the RIM8 dataset, we found that more than 95% of the resamples had a lower rate than the original data set. This finding supplies evidence for a temporal signal in this lineage (Figure 1). We did not detect a temporal signal in RIM12_C and RIM44; however, those data sets contain genomes from three time points only, which is likely insufficient temporal information for the estimation of evolutionary rates. The method of root-to-tip (RTT) correlation gives consistent results where only the RIM8 masked alignment contains a high temporal signal (RTT r^2^ > 0.5) (Figure S1, Table S1). In particular, none of the lineages where the recombination signal was not eliminated have a high temporal signal; thus, recombination masking seems to be a prerequisite for the temporal signal. We thus conclude that, from the three analyzed lineages, where substitutions could be separated from recombination events, only lineage RIM8 shows a temporal signal for substitutions. Thus, the following analysis is restricted to that lineage.

### 3.3. RIM8 Evolutionary Rates

**Figure 2:**
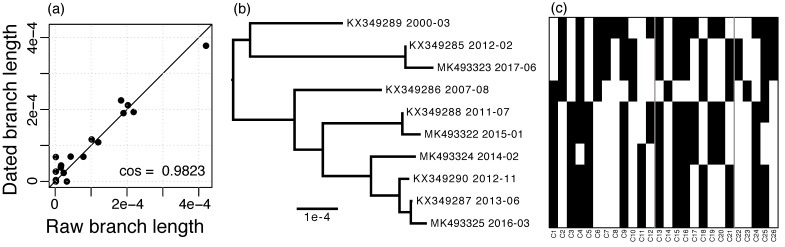
RIM8 dating. (**a**) Agreement of raw branch lengths and branch lengths dated with LSD, straight line gives the identity; (**b**) Dated phylogeny estimated with LSD, accessions, and sampling dates are given; (**c**) Presence–absence matrix of variable genes, rows are ordered by the phylogeny shown in (**b**) and columns are ordered by alignment position (see also Table S2); the three regions of variable genes are separated by gray lines.

The RIM8 whole-genome alignment has a length of 173,430 nt and contains 3937 (2.3%) variable positions, of which 152 (3.9%) are multiallelic. In contrast, the masked alignment is of length 124,593 nt and contains only 160 (0.13%) variable positions, of which none are multiallelic. Using the masked alignment, a substitution rate of 1.275 × 10^−5^ substitutions per site per year was estimated. The dated phylogeny shows a strong agreement with the raw branch lengths, which supports that the substitutions in this lineage follow a molecular clock (Figure 2a,b).

**Figure 3:**
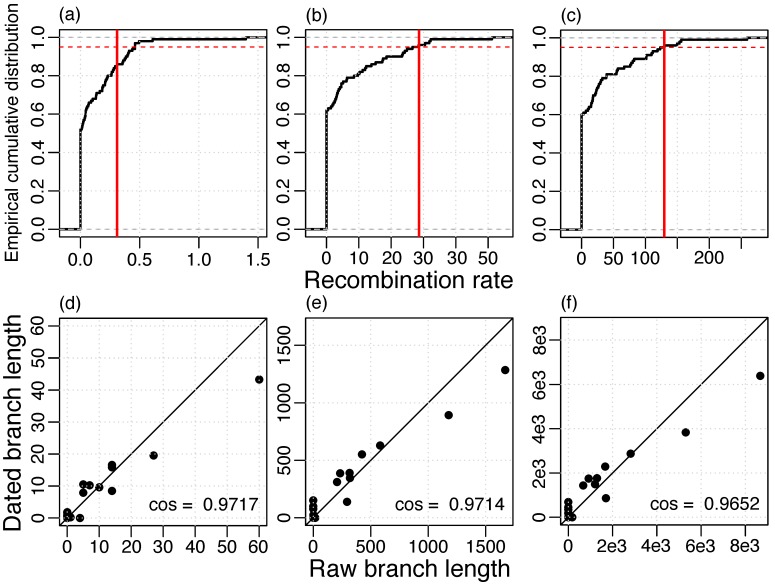
Temporal signal of recombination rate estimation (**a**,**d**) events, 0.3096 recombinations/year; (**b**,**e**) alterations, 28.64 nucleotide alterations/year; (**c**,**e**) lengths, 129.5 nucleotides transferred/year; (**a**–**c**) Vertical red line: Estimate of the real data set, 95% of the resampled data sets lie below the red dashed line; (**d**–**f**) Agreement of raw branch lengths and branch lengths dated with LSD, straight line gives the identity.

To estimate the rate of recombination, we reconstructed recombination events based on the whole-genome alignment and the ML phylogeny of the masked alignment. Then, the recombination rate was estimated using LSD with phylogenies where branch lengths represent recombinations. We found a strong agreement between the raw branch lengths and the dated branch lengths and, in addition, a high temporal signal for different measures of recombination (Figure 3). Notably, a higher temporal signal was estimated when recombination was measured in numbers of nucleotide alterations (Figure 3b) or in total length of the transferred segments (Figure 3c) compared to number of events (Figure 3a). Thus, the recombination detection method might infer the correct regions of recombination, but not necessarily the correct recombination boundaries, i.e., it might split true events into multiple segments or merge different events into a single event. Those inaccuracies can lead to an incorrect estimation of the number of events, but have a small effect on the total transferred segment length and the number of nucleotide alterations.

### 3.4. Variable Gene Families

The RIM8 lineage includes 234 homologous protein families, of which 208 are core families, i.e., they are present in each genome, and 26 are variable gene families, i.e., they are present in less than 10 genomes (Figure 2c; see accessions in Table S2). No paralogs were observed.

**Figure 4:**
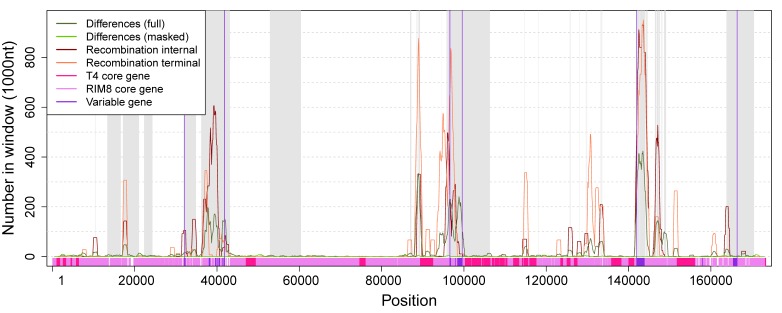
Numbers of differences and positions in recombination events along the whole-genome alignment. Regions shaded in gray are absent in the masked alignment. Numbers of differences and recombination positions are shown for windows of size 1000 with an offset of 100. Variable genes cluster in three regions (marked by vertical violet lines). Region 1: 32,085—41,835, Region 2: 96,595–99,589, Region 3: 141,976–166,384. The whole alignment contains 11,837 (6.8%) positions that are involved in recombination events. Each variable region contains a significantly higher proportion of recombination positions (Fisher’s exact test, *p*-value < 10^−6^): Region 1: 1961 (20%), Region 2: 1027 (34%), Region 3: 3582 (15%).

To determine whether some regions of the genome contain more differences, or are more affected by recombination and gene content variation, those events were mapped to the whole genome alignment (Figure 4, Table S2). We observe that variable genes cluster in three regions, where Region 2 corresponds to the previously identified hyperplastic region [21]. Our analysis reveals that variable genes co-localize with recombination events (Figure 4), which suggests that gene content variation is mediated by recombination. Except for C1 and C2, all variable proteins have homologs in other cyanophages. Most variable proteins are annotated as “hypothetical protein”, whereas only two of the variable proteins had a functional annotation and both are located in the hyperplastic region (Table S2). C15 is annotated as plastocyanin (PetE), which functions in the photosynthesis machinery as an electron transporter [41]. Plastocyanin has been previously described as a “sporadically distributed” AMG in cyanophages [22,42]. It has been reported that phage PetE sequences form a distinct clade that is separated from bacterial PetE and show phage-specific differences; thus, the phage protein might perform a different function [9]. C18 is annotated as 30S ribosomal protein S6 glutaminyl transferase (RimK family). RimK is known to modulate the SOS response in *Escherichia coli* [43] and it has been suggested to be involved in the response to oxidative or UVA stress in cyanobacteria [44]. Thus, C15 and C18 are homologs of cyanobacterial genes and their functional annotation suggests that their expression may be beneficial for the host metabolism (i.e., the phage) during an infection.

## 4. Discussion

**Figure 5:**
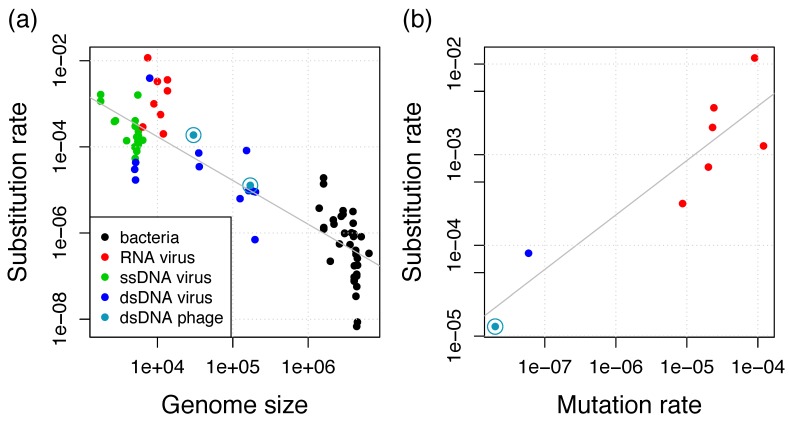
(**a**) Negative relationship between substitution rate and genome size for viruses and bacteria. Additional data points from the collections in References [17,48] and additional estimates from Reference [46] if they are also in (**b**); (**b**) Positive relationship between substitution rate and mutation rate for viruses. Additional data points from References [13,46]. Gray lines show the linear regressions.

Here, we estimated genome-wide evolutionary rates in marine cyanophages of the family *Myoviridae*. The estimated substitution rate of 1.3 × 10^−5^ substitutions per site per year is about tenfold lower in comparison to the rate we previously estimated from a lineage of dairy *Siphoviridae* phages (1.9 × 10^−4^ substitutions per site per year) [18]. Both rates are substantially higher than bacterial substitution rates; rather, they are in the range of rates of eukaryotic viruses (Figure 5a).

The per-base mutation rate has been shown to scale negatively with the genome size and with the effective population size N_e_ [45]. In eukaryotes, the population size is considered as the major determinant of mutation rates, whereas the genome size is considered as the main determinant in microorganisms. This inverse correlation between mutation rate and genome size has been initially shown for bacteria and bacteriophages and is now known as Drake’s rule [12]. For slowly-evolving viruses, including dsDNA viruses, the substitution rate increases linearly with the mutation rate [46] and we find that this relationship is also supported by including the T4 mutation rate of 2 × 10^−8^ mutations per site per cell infection [12], which is potentially very similar to the RIM8 mutation rate (Figure 5b). This relationship supplies evidence for the neutral theory of molecular evolution, i.e., most mutations are neutral or deleterious such that substitutions observed in genomic data are neutral or nearly neutral [47]. Under the neutral theory, the substitution rate is proportional to the mutation rate by a factor which is the product of the number of generations (cell infections per year) and the fraction of effectively neutral mutations [46]. Thus, viral and microbial substitution rates seem to be determined by Drake’s rule, which determines the mutation rate, and the neutral theory, which determines the factor of the mutation to the substitution rate. This leads to the previously observed negative relationship between substitution rate and genome size for eukaryotic viruses and bacteria [17,46]. Our results reveal that bacteriophages also conform to this relationship and that the difference between both phage estimates can be well explained by the difference in genome length (Figure 5a). We thus conclude that phage substitution rates also conform to Drake’s rule and the neutral theory.

Adaptation to resistant hosts has been shown experimentally in RIM8 [49]. Since the adaptation to sub-optimal hosts is expected to increase the evolutionary rate [11], it is expected to result in a non-homogenous substitution rate. In contrast, we observe a strong temporal signal, which is evidence for a homogeneous substitution rate over the sampling time. This suggests that adaptation to sub-optimal hosts over part of the sampling time did not modify the substitution rate. We thus conclude that adaptations to sub-optimal hosts are transient and did not contribute significantly to genome evolution in the RIM8 lineage.

Furthermore, we observe a high relative frequency of recombination to mutation in bacteriophage lineages (Table 2). Thus, the contribution of horizontal evolution exceeds the contribution of vertical evolution by multiple folds. Consequently, we conclude that horizontal processes are important drivers of bacteriophage genome evolution. In addition to the relative contribution, absolute recombination rates in the number of events, nucleotide alterations, and the transferred length per year have been reported for two independent bacteriophage lineages (this study and Reference [18]). We thus conclude that a molecular clock of substitution and of recombination acts on phage short-term evolution. Furthermore, we find that the phage r/m estimates exceed the r/m estimates from bacterial organisms (Table 2). The comparison is restricted to genome-wide r/m estimates because they were observed to be higher than r/m estimates from multilocus sequence typing [50]. No genome-wide recombination rate estimates for marine *Synechococcus* are available; thus, the RIM8 r/m cannot be compared to the host directly. Nevertheless, by comparing a range of bacterial and phage estimates, we conclude that the contribution of recombination to mutation is generally higher in phages. However, the factors that determine phage recombination rates cannot be assessed based on only two data sets.

The analysis of five cyanophage clusters and additional subclusters resulted in a single lineage where the recombination detection was successful and the temporal signal was sufficient. In six of the nine data sets, the applied recombination detection method could not distinguish between recombination and substitution, which prevented their further analysis. Future development of recombination detection methods that are designed for phage genomes can potentially improve this step and allow for the reanalysis of the data sets presented here. Additionally, a large number of sampling time points is important, as, among the three data sets where the recombination detection was successful, two datasets contain samples from only three time points and were accordingly estimated to have an insufficient temporal signal. Further resolution of evolutionary rates and the determinants of phage recombination rates require increased sample sizes of additional phage lineages.

Variable phage genes typically encode non-essential proteins that are involved in host recognition or that are AMGs that modulate the host metabolism during an infection [21,55]. AMGs are especially well studied in cyanophages (see reviews [4,5,9,56]). Cyanophage fitness during an infection can be increased by phage-encoded components of the photosystems [7,56,57], as shown, for example, for psbA [58]. AMG content varies between phage genomes and this variation is influenced by differing selection pressures among environments [22,59]. In our analysis of RIM8, functions could be predicted for only two of the 26 variable genes and both predictions indicate that they are auxiliary genes that are involved in the host metabolic processes. Due to the low absolute number of variable genes in bacteriophage genomes, estimated rates of gene turnover would be unreliable. We observe that gene content variation is mediated by recombination, which is consistent with previous observations for T4-like phages [26]. Thus, although independent gene turnover rates cannot be estimated, the magnitude of the recombination rate can be used as an alternative indicator for the gene turnover rate. The high recombination rates observed here also support that gene transfer mediated by recombination is an important contributor to cyanophage evolution.

## Figures and Tables

**Table 1:** Data sets overview.

Data Set	Number of Genomes	Time Span (Years)	Number of Timepoints	Mean Length (nt)	*p*-Value Phi Test	r/m
RIM2	59	8	9	175,301	6.01 × 10^−34^	2.156
RIM2_A	47	8	8	175,310	3.94 × 10^−27^	1.574
RIM2_B	10	1	4	175,309	5.24 × 10^−9^	3.385
RIM8	10	17	10	170,485	**0.0151**	7.833
RIM12	21	13	9	174,726	5.98 × 10^−10^	8.421
RIM12_A	10	13	7	174,271	2.07 × 10^−12^	7.053
RIM12_C	7	1	3	175,605	**0.013**	15.37
RIM14	9	2	6	179,756	9.79 × 10^−8^	2.214
RIM44	8	7	3	195,353	**0.059**	0.2884

Classification of genomes into clusters and subclusters according to Reference [29]. Only clusters and subclusters with at least six genomes are included in the analysis. Phi test *p*-values > 0.01 are marked in bold.

**Table 2:** Typical estimates of r/m from the literature. The table is restricted to genome-wide r/m estimates.

Name	Group	r/m	Reference
*Staphylococcus aureus*	Bacteria	0.283	[36]
*Pseudomonas aeruginosa*	Bacteria	0.853	[51]
*Escherichia coli*	Bacteria	1.024	[52]
*Salmonella enterica*	Bacteria	1.14	[53]
*Bacillus cereus*	Bacteria	3.4	[54]
*Streptococcus pneumonia*	Bacteria	7.2	[50]
Cyanophage RIM8	dsDNA phage	7.8	This study
936 group of phages	dsDNA phage	23.5	[18]

## Supplementary Materials

The following are available online at https://www.mdpi.com/1999-4915/11/8/720/s1, Figure S1: Plot of root-to-tip distance against isolation date; Table S1: Temporal signal; Table S2: Protein families.
