# Supplementary material for: Rates of Molecular Evolution in a Marine Synechococcus Phage Lineage
Source: Viruses. 2019 Aug 6;11(8):720. doi: 10.3390/v11080720 (PMC6722890; doi:10.3390/v11080720)
Supplement: Supplementary file 1 [file viruses-11-00720-s001.zip › Supplement.pdf]

Supplementary Material

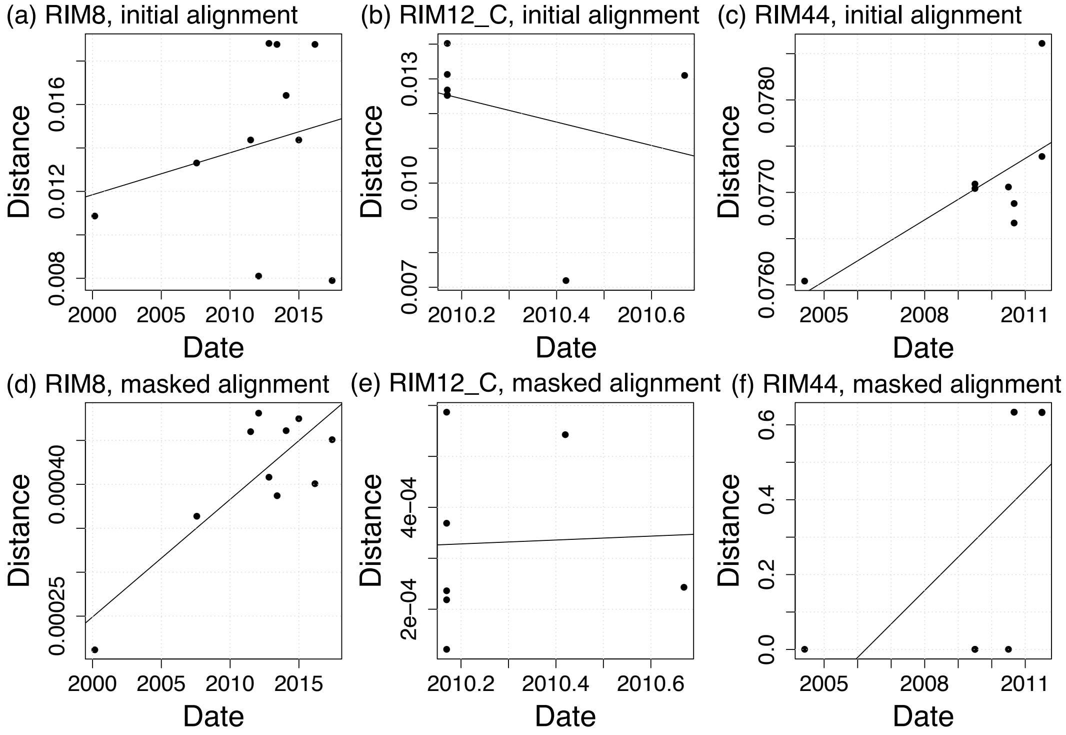

Figure S1. Plots of root-to-tip distance against isolation date. The line shows the linear regression.

Table S1. Temporal signal.

		Initial alignment				Masked alignment			
Data set	p-value Phi test	Length	RTT r2	RTT rate	LSD rate	Length	RTT r2	RTT rate	LSD rate
RIM2	6.01E-34	176619	0.007402	2.69E-04	1.00E-10	145832	0.005428	4.80E-06	7.67E-06
RIM2_A	3.94E-27	176002	0.007044	-5.79E-05	1.00E-10	156444	0.000145	8.93E-07	1.00E-10
RIM2_B	5.24E-09	175809	0.04372	4.78E-04	9.42E-04	128222	0.180924	6.50E-05	4.48E-05
RIM8	**0.0151**	173430	0.05296	1.93E-04	6.08E-05	124593	**0.686509**	1.34E-05	1.28E-05
RIM12	5.98E-10	184878	0.021512	1.56E-03	1.00E-10	100902	0.001547	4.70E-06	4.91E-06
RIM12_A	2.07E-12	176026	0.002011	5.13E-05	2.56E-06	159097	0.000028	6.36E-07	1.00E-10
RIM12_C	**0.013**	179187	0.086151	-3.37E-03	1.00E-10	161566	0.001897	3.89E-05	2.07E-06
RIM14	9.79E-08	181443	0.167003	-3.02E-03	1.00E-10	179994	0.209073	-5.40E-04	1.00E-10
RIM44	**0.059**	207024	0.486262	2.21E-04	2.54E-04	43429	0.367625	8.94E-02	1.00E-10

Phi test p-values > 0.01 and r^2 values >0.5 are marked in bold. RTT – root-to-tip.

Table S2. Protein families. (separate file)

Start and end give the location of the protein families in the full alignment. Type denotes *T* for a T4 core gene or the number of the family for a variable gene. Annotation is deduced from the protein product annotation in the cyanophage GenBank files and, for T4 core genes, from the notes in the T4 GenBank file.
